# Reorganization of Extracellular Matrix in Placentas from Women with Asymptomatic Chagas Disease: Mechanism of Parasite Invasion or Local Placental Defense?

**DOI:** 10.1155/2012/758357

**Published:** 2011-10-05

**Authors:** Juan Duaso, Erika Yanez, Christian Castillo, Norbel Galanti, Gonzalo Cabrera, Gabriela Corral, Juan Diego Maya, Inés Zulantay, Werner Apt, Ulrike Kemmerling

**Affiliations:** ^1^Programa de Anatomía y Biología del Desarrollo, Instituto de Ciencias Biomédicas, Facultad de Medicina, Universidad de Chile, Independencia, Región Metropolitana, 1027 Santiago de Chile, Chile; ^2^Programa de Biología Celular y Molecular, Instituto de Ciencias Biomédicas, Facultad de Medicina, Universidad de Chile, Independencia, Región Metropolitana, 1027 Santiago de Chile, Chile; ^3^Servicio de Obstetricia y Ginecología, Hospital de Illapel, Independencia, IV Región, 0512 Illapel, Chile; ^4^Programa de Farmacología Molecular y Clínica, Instituto de Ciencias Biomédicas, Facultad de Medicina, Universidad de Chile, Independencia, Región Metropolitana, 1027 Santiago de Chile, Chile; ^5^Departamento de Estomatología, Facultad de Ciencias de la Salud, Universidad de Talca, Avenida Lircay s/n, VII Región, 3460000 Talca, Chile

## Abstract

Chagas disease, produced by the protozoan *Trypanosoma cruzi* (*T. cruzi*), is one of the most frequent endemic diseases in Latin America. In spite the fact that in the past few years *T. cruzi* congenital transmission has become of epidemiological importance, studies about this mechanism of infection are scarce. In order to explore some morphological aspects of this infection in the placenta, we analyzed placentas from *T. cruzi*-infected mothers by immunohistochemical and histochemical methods. Infection in mothers, newborns, and placentas was confirmed by PCR and by immunofluorescence in the placenta. *T. cruzi*-infected placentas present destruction of the syncytiotrophoblast and villous stroma, selective disorganization of the basal lamina, and disorganization of collagen I in villous stroma. Our results suggest that the parasite induces reorganization of this tissue component and in this way may regulate both inflammatory and immune responses in the host. Changes in the ECM of placental tissues, together with the immunological status of mother and fetus, and parasite load may determine the probability of congenital transmission of *T. cruzi*.

## 1. Introduction

American Trypanosomiasis, or Chagas disease, is a zoonosis caused by *Trypanosoma cruzi* (*T. cruzi*). Currently, 10 million people in the Americas, from Mexico in the north to Argentina and Chile in the south, are estimated to be infected [1]. For thousands of years, Chagas disease was known only in the Region of the Americas, mainly in Latin America, where it has been endemic [2]. In past decades, it has been increasingly detected in other non-endemic countries in the American (Canada and the United States), the Western Pacific (mainly Australia and Japan) and the European continents. The presence of Chagas disease outside Latin America is the result of population mobility, notably migration, but cases have been reported among travelers returning from Latin America and even in adopted children [3]. Subsequent transmission occurs through transfusion, vertical, and transplantation routes [3]. 

The vertical transmission of *T. cruzi *cannot be prevented, but early detection and treatment of congenital infection achieves cure rates close to 100 per cent [4].

Fetal and maternal tissues are separated by a fetal epithelium (the trophoblast), the greatest area of which is in the villous placenta, the site of nutrient and gas exchange [5]. The human placenta is classified as a hemochorial villous placenta in which the free chorionic villi are the functional units. These chorionic villi are formed by the trophoblast and the villous stroma. The trophoblast is formed by a single multinucleated cell layer (syncytiotrophoblast) which contacts maternal blood in the intervillous space, and by the cytotrophoblast which contains replicating progenitor cells. The trophoblast is separated by a basal lamina from the villous stroma, which is connective tissue containing vascular endothelium, fibroblasts, and macrophages [5]. Trophoblast, basal laminae, and villous stroma with endothelium of fetal capillaries form the placental barrier that must be crossed by different pathogens, including *T. cruzi*, in order to infect the fetus during vertical transmission [6, 7]. 

Damage to the villous placenta is almost always accompanied by inflammation, either in the intervillous space or within the fetal villi, and in severe cases is accompanied by loss of the protective trophoblast. Extensive placental damage may lead to fetal loss, intrauterine growth retardation [5], and clinical manifestations that can be observed in acute congenital Chagas disease [8, 9].

We have previously demonstrated that *T. cruzi *induces syncytiotrophoblast destruction and detachment in chorionic villi explants, and selective disorganization of basal lamina and disorganization of collagen I in the connective tissue of villous stroma [6]. However, no studies observing similar histopathological alterations in the different tissue compartments of chorionic villi from placentas of mothers with Chagas disease have been reported. 

The aim of the present study was to determine whether the tissue disorganization and destruction of chorionic villi, described previously in *ex vivo* infected chorionic villi explants, can also be observed in placentas from Chagasic women. The diagnosis of Chagas disease in the women studied was performed by standard serological testing and PCR. Additionally, the parasite was detected in the placenta by immunofluorescence and PCR. Histopathological analysis, immunohistochemical studies of basal lamina, and histochemical studies of carbohydrate-rich molecules and collagen I in villous stroma of these placentas were performed.

## 2. Material and Methods

### 2.1. Patients and Diagnosis of T. cruzi Infection

**Figure 1:**
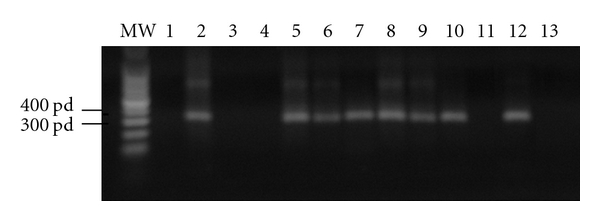
*Detection of T. cruzi in mother, newborn, and placenta by PCR*: A 330-base pair fragment of the *T. cruzi* satellite DNA was amplified as described in Material and Methods. MW: molecular marker, lane 1: Negative control without DNA; lane 2: DNA from *T. cruzi* epimastigotes; lane 3: noninfected human blood DNA; lane 4 non-infected human placenta DNA; lanes 5–7: parasite DNA detected in peripheral blood (lane 5), in placenta (lane 6), and respective umbilical chord from neonate (lane 7) of mother 1; lanes 8–10: parasite DNA detected in peripheral blood (lane 8), in placenta (lane 9), and respective umbilical cord from neonate (lane 10) of mother 2; lanes 11–13: parasite DNA detected in peripheral blood (lane 11), in placenta (lane 12) and respective umbilical chord from neonate (lane 13) of mother 3. Note that in mother 3 parasite DNA can only be detected in the placenta. The PCR product was subjected to electrophoresis in 1.6% agarose gels and stained with ethidium bromide. PCR markers from Promega were employed as molecular weight standards.

Three pregnant women with asymptomatic Chagas disease were enrolled from the hospitals of Illapel, Servicio de Salud de Coquimbo, IV Region of Chile. Informed consent for the experimental use of the placentas was given by each patient as stipulated by the Code of Ethics of the Faculty of Medicine of the University of Chile. Maternal *T. cruzi* infection was assessed by standard serological techniques and PCR (Figure 1(a) and Table 1). *T. cruzi* infection in neonates was diagnosed by detection of parasites in umbilical cord or in peripheral blood by microscopic examination using heparinized microhematocrite tubes and PCR (Figure 1(a) and Table 1). All of the neonates were negative for the presence of the parasite by direct parasitological examination. Two mothers and their respective newborns were positive for parasite DNA detection by PCR (Figure 1 lines 5 & 7 and 8 & 10), the third mother and her newborn were negative for parasite DNA detection by PCR (Figure 1 lines 11 & 13). Nevertheless, this last mother was positive using standard serological techniques. Gestational age was determined by clinical and ultrasound analysis. Clinical data of the mothers and newborns are shown in Table 1. 

For parasite DNA detection by PCR, a 330-base pair fragment of the *T. cruzi* satellite DNA was amplified as described previously [10]. The sequence of the oligonucleotides is the following: forward (5-AAATAATGTACGG(T/G)GAGATGCATGA-3, specific primer 121) and backward (5-GGTTCGATTGGGGTTGGTGTAATATA-3 specific primer122). The PCR product was subjected to electrophoresis in 1.6% agarose gels and stained with ethidium bromide. PCR markers from Promega were employed as molecular weight standards [10].

### 2.2. Placentas

We used three placentas from asymptomatic chagasic women. Placentas from control healthy women were obtained from vaginal or caesarean deliveries of uncomplicated pregnancies from the Hospital San José, Servicio de Salud Norte; Region Metropolitana, Santiago, Chile. Exclusion criteria for the control placentas were the following: major fetal abnormalities, placental tumor, intrauterine infection, obstetric pathology, and any other maternal disease. Villous tissue was obtained from the central part of cotyledons and immediately fixed in 10% formaldehyde in 0.1 M phosphate buffer (pH 7.3) for 24 hours.

### 2.3. Histological and Histochemical Techniques

The fixed tissues were dehydrated in alcohol, clarified in xylene, embedded in paraffin, and sectioned at 5 *μ*m. Paraffin-embedded histological sections that were stained with hematoxylin-eosin for routine histological analysis, Picrosirius red-hematoxylin [6] and Arteta [11] for collagen histochemistry, and periodic acid-Schiff (PAS) for carbohydrate containing tissue elements (Sigma-Aldrich kit 395B) were applied. As a control of PAS method, 5 *μ*m-thick sections were incubated with a 4 *μ*g/mL solution of *α*-amylase (Nutritional Biochemical Corporation) in PBS pH 6.0, for 30 minutes at 37°C prior to the PAS reaction (data not shown). A decrease in the intensity of the PAS stain reaction after the enzyme treatment was considered as evidence for the presence of glycosylated molecules [11].

### 2.4. Immunohistochemistry

The placental chorionic villi were fixed, dehydrated, and embedded in paraffin as described above. Standard immunoperoxidase techniques were used to show collagen IV, heparan sulphate and fibronectin. The primary antibodies were applied individually to each section at 4°C overnight (anti-collagen IV Novocastra NCL-COLL-IV, dilution 1 : 100 v/v; anti-heparan sulphate Novocastra NCL-CD44-2, dilution 1 : 40 v/v; anti-fibronectin ABR MA1-83176, dilution 1 : 50 v/v). Immunostaining was performed using a horseradish peroxidase-labelled streptavidin biotin kit (RTU-Vectastain kit) following the manufacturer's directions using diaminobenzidine as the chromogen. Sections were counterstained with Mayer's haematoxylin (DAKO) and mounted with Entellan (Merck). Immunohistochemical controls were performed by replacing the primary antibodies with phosphate buffered saline. All controls were negative. All sections were examined by light microscopy (Leitz Orthoplan), and images were captured with a Canon 1256 camera.

### 2.5. Detection of T. cruzi in Placental Tissue

*T. cruzi* invasion was observed by immunofluorescence and by parasite DNA detection using the polymerase chain reaction (PCR).

**Immunofluorescence**

The placental chorionic villi were fixed, dehydrated, and embedded in paraffin as described above. A monoclonal antibody (mAb 25, dilution 1 : 100 v/v) specific for the *T. cruzi* flagellar calcium-binding protein (a gift from Dr. Schenkman, Universidade de Sao Paulo, Sao Paulo, Brasil) was applied to each section overnight at 4°C. The preparations were washed with PBS and incubated with anti-mouse IgG conjugated with fluorescein (ScyTek, ACA) in the presence of 1 *μ*g/mL of 4′6′diamidino-2-phenylindole (DAPI). Afterwards, sections were mounted in VectaShield (ScyTek, ACA) and observed in a Nikon Eclipse E400 microscope (Tokio, Japan).

**PCR**

Genomic DNA that was extracted from the paraffin included placental tissue with the NucleoSpin Tissue kit (Machery-Nagel) according manufacturer's instructions. The same satellite DNA fragment of *T. cruzi*, used for parasite detection in blood of mother and child (see above), was amplified.

## 3. Results

### 3.1. T. cruzi Detection in Mothers, Newborns, and Placentas

The diagnosis of Chagas disease of the three mothers enrolled in our study was performed by standard serological techniques. After delivery, blood samples from the mothers and umbilical cords from respective neonates were processed for parasite DNA detection by PCR (Figure 1 lines 5 & 7 (mother & neonate 1), 8 & 10 (mother & neonate 2), 11 & 13 (mother & neonate 3)). DNA from the placentas was obtained from formalin-fixed placental tissue samples (Figure 1 lines 6, 9 and 12 show the results from placentas from mother 1, 2, and 3, resp.). Interestingly, one of the mothers (mother 3) and her newborn were negative for PCR (Figure 1 lines 11 and 13) but the placenta was positive (Figure 1 line 12). The negative result for parasitic DNA detection by PCR in this mother and newborn may be due to a very low parasitemia, characteristic in the asymptomatic and chronic phases of Chagas disease. In these phases, the sensitivity of the techniques depends on the varying concentration of parasites in blood [1, 10]. For this reason, negative results do not necessarily indicate a lack of parasites in the blood or absence of infection. Serological diagnosis of *T. cruzi* infection is more sensitive than PCR [10, 12]; all three mothers analyzed by us had positive serology.

**Figure 2:**
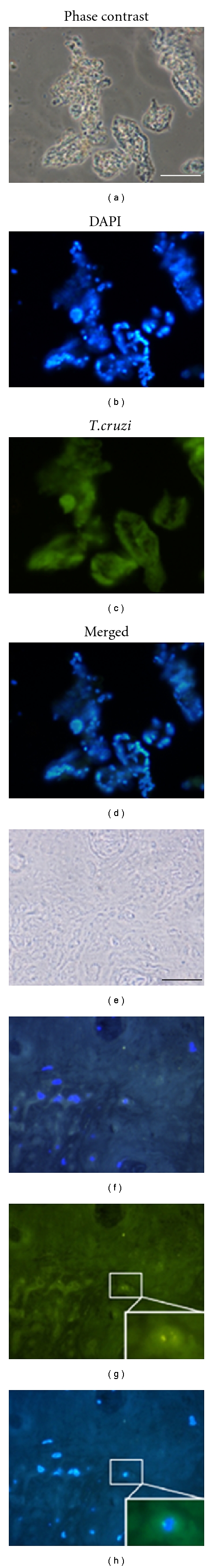
*Detection of T. cruzi in human placental chorionic villi from infected mothers*. The presence of parasites in villous stroma of chorionic villi from *T. cruzi* infected placentas was shown by immunofluorescence, detected using mAb 25 antibodies. In (a–d), noninfected chorionic villi (controls) are shown. Panels (a–d) and (e–h) show images of the same fields. Panels (a) and (e): phase contrast; (b) and (f): nuclear staining with DAPI; (c) and (g): detection of the parasite by immunofluorescence; (d) and (h): merged images of (b-c) and (f-g), respectively. Inserts show an amplified region of the detection of the parasite by immunofluorescence (g-h). Bar scale (a–d): 50 *μ*m; (e–h): 25 *μ*m.

Figure 2 shows the detection of the parasite by immunofluorescence. The parasite can be visualized in connective tissue cells of the villous stroma (Figures 2(e)–2(h)). Placentas from healthy, non-*T. cruzi*-infected, women did not show evidence of the presence of the parasite (Figures 2(a)–2(d)). Interestingly, parasites do not form amastigote nests (the obligate intracellular replicative form of the parasite) as seen in other tissues like the myocardium [13, 14]. In the placentas of *T. cruzi*-infected women, only few parasites can be visualized. The limited presence of the parasite maybe due to a very low parasitemia present in the asymptomatic or chronic phases of the disease. Another explanation for this fact may be the presence of a local placental antiparasitic mechanism, like the increase of nitric oxide which has anti-trypanosomal activity [15]. We observed the same limited presence of the parasite in the *ex vivo* infected explants of chorionic villi [6].

### 3.2. Histopathological Analysis of Placentas from Women Infected with T. cruzi

**Figure 3:**
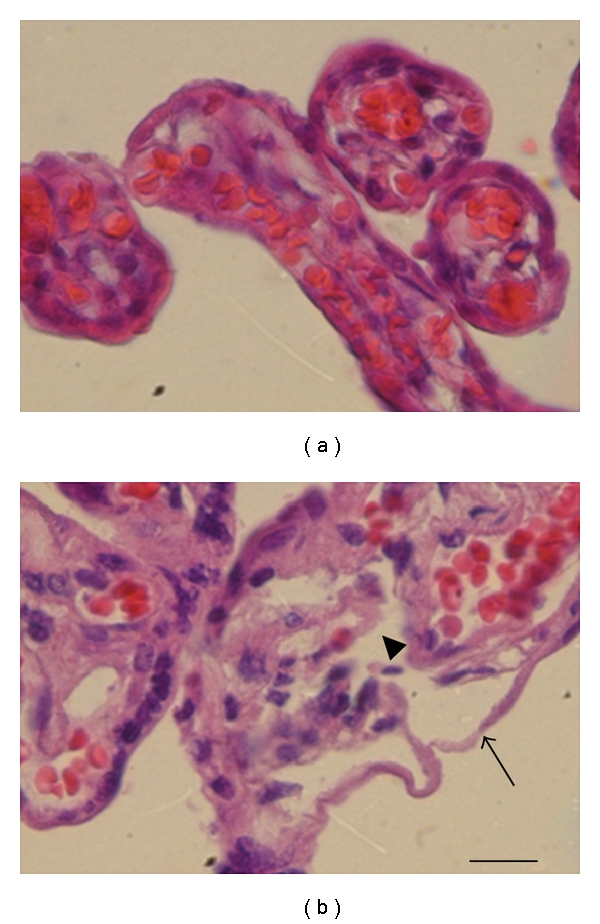
*Histopathological analysis of placentas from mothers infected with T. cruzi*. Chorionic villi from *T. cruzi*-infected placentas (b) show severe tissue damage compared to control villi from healthy noninfected woman (a). Detachment and disorganization of the syncytiotrophoblast (arrow) as well as fetal connective tissue destruction of the villous stroma (arrowhead) is observed. Chorionic villi were processed for routine histological techniques and stained with hematoxylin-eosin. Bar scale: 25 *μ*m.

Figure 3(b) shows severe tissue disorganization and destruction of the chorionic villi from *T. cruzi*-infected mothers, detachment of the syncytiotrophoblast (arrows), and destruction of villous stroma (arrowhead). No inflammatory infiltrate is observed in placentas from infected women or control placentas. The control placentas from healthy women show an intact structure of the chorionic villi (Figure 3(a)).

### 3.3. Placental Chorionic Villi from Women Infected with T. cruzi Present Disorganization of the Extracellular Matrix

**Figure 4:**
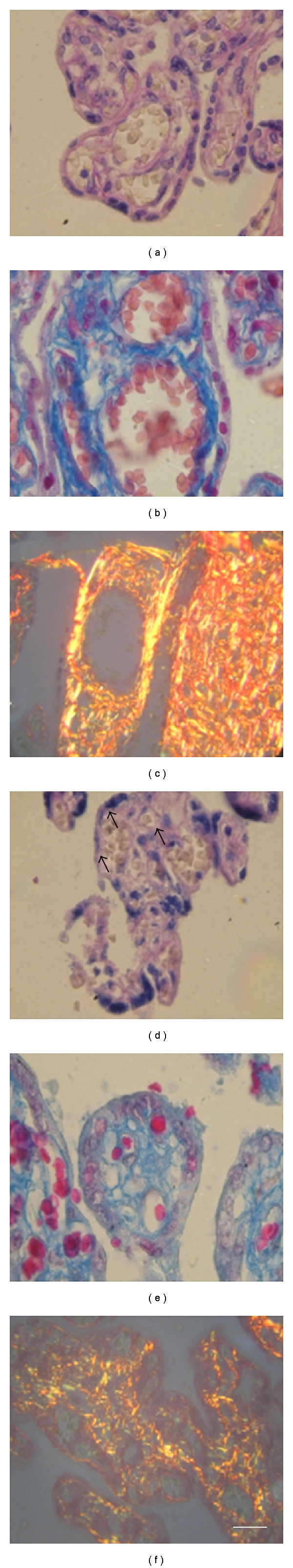
*Placentas from mothers infected with T. cruzi present severe disorganization of chorionic villi ECM*. Placental tissue from healthy noninfected (a–c) and infected (d–f) women were processed for routine histochemical methods and stained with the PAS method for the detection of glycosylated components (a, d) with the Arteta trichromic (b, e), and picrosirius red-hematoxylin (c, f) methods for collagen histochemistry. Placental chorionic villi from infected mothers show reduced staining of glycosylated molecules (d), especially in basal lamina (arrows) compared to controls (a). Samples from infected mothers also show reduced histochemical reaction for collagen in basal lamina (e) and in villous stroma (f) compared to controls (b, c). Bar scale: 20 *μ*m.

The extracellular matrix (ECM) is the noncellular component present in all tissues and organs and not only provides essential physical scaffolding for the cellular constituents, but also initiates crucial biochemical and biomechanical cues that are required for different tissue functions [16]. The ECM is composed of collagens, elastin, proteoglycans (including hyaluronan), and noncollagenous glycoproteins forming a complex, three-dimensional network among the cells of different tissues [17]. The non-collagenous proteins and proteoglycans are highly glycosylated and especially abundant in basal lamina [6, 18]. Several lines of evidence have shown that *T. cruzi *interacts with host ECM components producing breakdown products that play an important role in parasite mobilization and infectivity [6, 19]. We used PAS reagent to analyze the non-collagenous component of the ECM. The placentas from Chagasic women (Figure 4(d)) show a significant decrease in PAS reactivity compared to control tissue (Figure 4(a)). This reduced PAS reactivity is particularly evident in basal lamina (Figure 4(d) arrows).

The principal fibrous components of chorionic villi connective tissue of the villous stroma are collagen fibers, particularly collagen I [5]. Collagen fibers form the “basic skeleton” of the ECM three-dimensional network. We performed two histochemical stains to analyze the collagen molecules: arteta staining for collagen in basal laminae (Figures 4(b) and 4(e)) and Picrosirius red-hematoxylin for collagen I (Figures 4(c) and 4(f)). Histochemical reaction for collagen was reduced in the placentas from mothers infected with the parasite (Figures 4(e)–4(f)), indicating destruction or disorganization of the collagen fibers present in basal lamina or villous stroma of the chorionic villi.

### 3.4. Placental Chorionic Villi from Women Infected with T. cruzi Present Selective Disorganization of Basal Lamina

**Figure 5:**
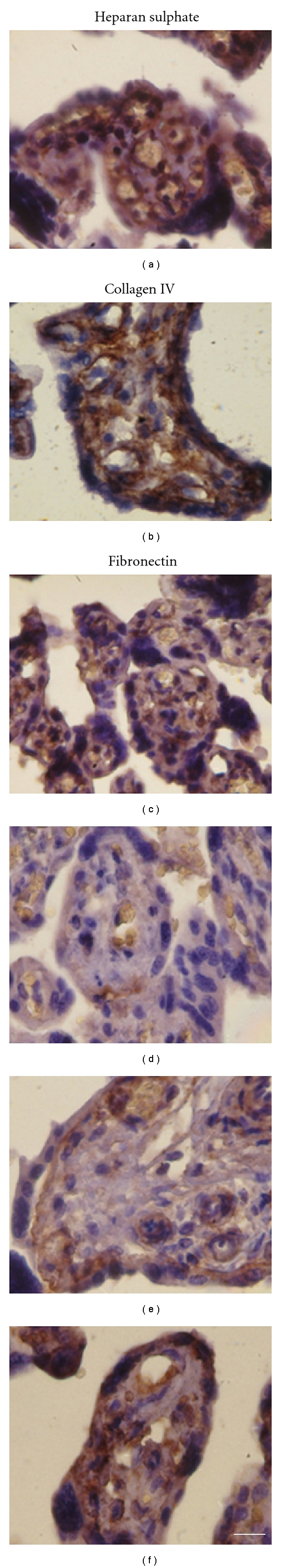
*Placentas from mothers infected with T. cruzi present selective basal lamina disorganization in chorionic villi*. Placental tissue from control non-infected (a–c) and infected (d–f) women were immunostained for heparan sulphate (a, d), collagen IV (b, e), and fibronectin (c, f). Placental chorionic villi from infected mothers completely lost their immunoreactivity for heparan sulphate (d) and showed reduced immunoreactivity for collagen IV (e), compared to controls (a, b). No difference in immunoreactivity for fibronectin was observed between controls and infected chorionic villi (c, f). Chorionic villi were processed for routine immunohistological techniques and counterstained with Mayer's hematoxylin. Bar scale: 20 *μ*m.

The basal lamina is part of the placental barrier that parasites have to cross in order to invade the fetal connective tissue of the villous stroma containing fetal capillaries. PAS and Arteta histochemical stains showed alteration in this specialized structure (Figures 4(d) and 4(e)). Collagen IV, heparan sulphate and fibronectin are components of the basal lamina [18]. We have previously shown that the parasite affects the immunoreactivity of heparan sulphate and collagen IV in *ex vivo* infected chorionic villi explants in a parasite concentration-dependent manner, and that fibronectin is not affected [6]. Placentas from *T. cruzi*-infected women show similar results; the immunoreactivity for heparan sulphate is loose or markedly decreased (Figure 5(d)). The immunoreactivity of collagen IV is decreased (Figure 5(e)) and no change in this parameter can be observed for fibronectin (Figure 5(f)) with respect to placenta from healthy women.

## 4. Discussion

During congenital *T. cruzi* infection, the parasite reaches the fetus by crossing the placental barrier [6–8]. Congenital Chagas disease is a product of a complex interaction between the maternal immune response, placental factors, and the parasite [20].

Congenitally infected newborns develop a parasite-specific T-cell immune response comparable to that of adults [21], as well as phenotypic and functional modifications of their NK cells [22]. On the other hand, newborns of *T. cruzi*-infected mothers are prone to produce higher levels of proinflammatory cytokines in comparison to those born to non-infected mothers [23]. From these observations, some authors postulated that some newborns from *T. cruzi*-infected mothers might naturally autocure their congenital infection [24]. Interestingly, the placentas from women with asymptomatic Chagas disease analyzed by us do not present inflammatory infiltrate. This aspect might represent an interesting aspect of the placental infection by *T. cruzi*, because of scarce invasion and/or destruction of the parasite within placenta. The lack of inflammation may be due to low parasite load in blood and tissues, characteristic of asymptomatic and chronic phases of the disease. Thus, other factors may also determine the capacity of the parasite to infect the placenta or, alternatively, impair the congenital transmission. 

Parasitemia is one factor associated with the risk of congenital transmission of the parasite. A high parasitemia, as can be detected in acute infection, correlates with a higher transmission rate of around 50%. In chronic infected patients, with very low parasitemia, the transmission rate is between 1 and 21% [25]. 

Recently, increasing evidence of the presence of local placental antiparasite factors has been reported. Among them, nitric oxide synthesis in placental tissue [15] and “heat-sensitive” agents [26] have been involved in local placental defense mechanisms. 

The ECM is another factor that should be considered during tissue invasion and pathogen infection. The ECM is a dynamic structure that interacts with cells and generates signals through feedback loops to control the behavior of cells. Thus, ECM macromolecules are bioactive and modulate cellular events such as adhesion, migration, proliferation, differentiation, and survival [27]. Additionally, ECM molecules are strictly organized, and this organization determines the bioactivity of the ECM. Even minor alterations such as a single amino acid substitution in a single ECM component can lead not only to altered physicochemical properties of tissues but also to changes in cellular phenotype and cell-matrix interactions [17]. It has been proposed that these changes in ECM structure and bioactivity in tissue function ultimately lead to development of disease [17]. It has been proposed that ECM alterations produced by *T. cruzi* not only promote its motility in tissues and its entrance into cells, but also alter the presence of cytokines and chemokines, which in turn permit *T. cruzi* to modulate and escape both the inflammatory response and the immune response [6, 19]. Alternatively, these changes in ECM function may be part of local placental defense mechanisms, which could explain why only very few parasites can be detected in the placenta. 

During tissue invasion, *T. cruzi* is able to interact with different elements of the ECM. Thus, the parasite presents surface molecules, such as gp85 [19] and gp83 [28], glycoproteins that bind to laminin and fibronectin [19, 28] as well as to sulphated glycosaminoglicans such as heparan sulphate [29]. The parasite can induce the expression of ECM molecules or decrease their presence [19]. The more obvious explanation for the decrease of ECM is that the parasite destroys the ECM by secretion of proteases like cruzipain [6, 30], which can degrade collagen I and IV [30], or by induction of matrix metalloproteases [31]. However, another possibility is that *T. cruzi* decreases the expression of these molecules by modulation of signal transduction pathways or cytoskeleton rearrangement. Probably, during tissue invasion *T. cruzi* first binds to molecules of the basal lamina, facilitating its internalization to the different cells in the underlying connective tissue. *T. cruzi* infection decreases fibronectin expression in cardiomyocyte culture. On the contrary, laminin protein expression levels do not change, but the distribution of this ECM component changes dramatically in this type of cell culture [32]. We have shown previously that in *ex vivo* infected human chorionic villi, the immunoreactivity for heparansulphate decreases or disappears completely in a parasite-concentration-dependent manner. Fibronectin does not present changes in its immunoreactivity or in its distribution pattern [6]. Here we confirm these results in placentas from mothers with asymptomatic Chagas' disease. In the *ex vivo* infection of chorionic villi, the immunoractivity for collagen IV decreases in the basal lamina between the trophoblast and villous stroma, but not around fetal capillaries [6]. In the placentas from the Chagasic women, collagen IV immunoreactivity is decreased in both basal laminae. It is probable that chronic exposure to the parasite during the entire pregnancy causes a more severe change in ECM than a 24-hour challenge in *ex vivo* infected placental tissue.

## 5. Conclusion

The evidence that placentas from *T. cruzi*-infected women present severe ECM alterations indicates that the parasite induces reorganization of this tissue component in such a way that may regulate inflammatory and immune responses in the host. If this is the case, changes in placental tissue ECM, together with the immunological status of mother and fetus, and parasite load may determine the probability of congenital transmission of *T. cruzi*.

## Figures and Tables

**Table 1:** Characterization of clinical features of mothers and neonates.

	Mother 1	Mother 2	Mother 3
Age mother (years)	43	32	39
Gestational age (weeks)	38	36	40
Type of delivery	Vaginal	Cesarean	Vaginal
Birth weight (g)	2880	2690	3550
APGAR 5 min	9.9	9.9	9.9
Chagas' disease diagnosis of the mother	ELISA/IFI +	ELISA/IFI +	ELISA/IFI +
Detection of the parasite in the neonate by direct parasitological examination	Negative	Negative	Negative
Detection of the parasite in the mother by PCR	Positive	Positive	Negative
Detection of the parasite in the neonate by PCR	Positive	Positive	Negative
Detection of the parasite in the placenta by PCR	Positive	Positive	Positive
